# Combined associations of family history and self-management with age at diagnosis and cardiometabolic risk in 86,931 patients with type 2 diabetes: Joint Asia Diabetes Evaluation (JADE) Register from 11 countries

**DOI:** 10.1186/s12916-022-02424-y

**Published:** 2022-07-14

**Authors:** Johnny T. K. Cheung, Eric Lau, Cyrus C. T. Tsui, Edmond L. N. Siu, Naomi K. W. Tse, Nicole Y. L. Hui, Ronald C. W. Ma, Alice P. S. Kong, Amy Fu, Vanessa Lau, Weiping Jia, Wayne H. H. Sheu, Leorino Sobrepena, K. H. Yoon, Alexander T. B. Tan, Yook-Chin Chia, Aravind Sosale, Banshi D. Saboo, Jothydev Kesavadev, Su-Yen Goh, Thy Khue Nguyen, Yotsapon Thewjitcharoen, Raymond Suwita, Andrea O. Y. Luk, Aimin Yang, Elaine Chow, Lee Ling Lim, Juliana C. N. Chan

**Affiliations:** 1 Faculty of Medicine, The Chinese University of Hong Kong, Shatin, Hong Kong Special Administrative Region, China; 2 Asia Diabetes Foundation, Shatin, Hong Kong Special Administrative Region, Shatin, China; 3 Department of Medicine and Therapeutics, The Chinese University of Hong Kong, Prince of Wales Hospital, Shatin, Hong Kong Special Administrative Region, China; 4 Hong Kong Institute of Diabetes and Obesity, The Chinese University of Hong Kong, Prince of Wales Hospital, Shatin, Hong Kong Special Administrative Region, China; 5 Li Ka Shing Institute of Health Sciences, The Chinese University of Hong Kong, Prince of Wales Hospital, Shatin, Hong Kong Special Administrative Region, China; 6 Shanghai Sixth People’s Hospital, Shanghai, China; 7 Department of Medicine, Taipei Veterans General Hospital, Taipei, Taiwan; 8 Heart of Jesus Hospital, San Jose City, Philippines; 9 Seoul St. Mary’s Hospital, College of Medicine, The Catholic University of Korea, Seoul, South Korea; 10 Department of Medicine, Faculty of Medicine, University of Malaya, Kuala Lumpur, Malaysia; 11 Department of Medical Sciences, School of Medical and Life Sciences, Sunway University, Subang Jaya, Selangor Malaysia; 12 Department of Primary Care Medicine, Faculty of Medicine, University of Malaya, Kuala Lumpur, Malaysia; 13 Diacon Hospital, Bangalore, India; 14 Dia Care - Diabetes Care & Hormone Clinic, Ahmedabad, Gujarat India; 15 Jothydev’s Diabetes & Research Center, Thiruvananthapuram, Kerala India; 16 Department of Endocrinology, Singapore General Hospital, Singapore, Singapore; 17 MEDIC Medical Centre, Ho Chi Minh City, Vietnam; 18 Diabetes and Thyroid Center, Theptarin Hospital, Bangkok, Thailand; 19 Cerebrocardiovascular Diabetes Group Clinic (CDG), Jakarta, Indonesia

**Keywords:** Age of diagnosis, Family history, Type 2 diabetes, Self-management, Cardiometabolic risks

## Abstract

**Background:**

Family history (FamH) of type 2 diabetes might indicate shared genotypes, environments, and/or behaviors. We hypothesize that FamH interacts with unhealthy behaviors to increase the risk of early onset of diabetes and poor cardiometabolic control.

**Methods:**

In a cross-sectional analysis of the prospective Joint Asia Diabetes Evaluation Register including patients from 427 clinics in 11 Asian countries/regions in 2007–2021, we defined positive FamH as affected parents/siblings and self-management as (1) healthy lifestyles (balanced diet, non-use of alcohol and tobacco, regular physical activity) and (2) regular self-monitoring of blood glucose (SMBG).

**Results:**

Among 86,931 patients with type 2 diabetes (mean±SD age: 56.6±11.6 years; age at diagnosis of diabetes: 49.8±10.5 years), the prevalence of FamH ranged from 39.1% to 85.3% in different areas with FamH affecting mother being most common (32.5%). The FamH group (*n*=51,705; 59.5%) was diagnosed 4.6 years earlier than the non-FamH group [mean (95% CI): 47.9 (47.8–48.0) *vs.* 52.5 (52.4–52.6), logrank *p*<0.001]. In the FamH group, patients with both parents affected had the earliest age at diagnosis [44.6 (44.5–44.8)], followed by affected single parent [47.7 (47.6–47.8)] and affected siblings only [51.5 (51.3–51.7), logrank *p*<0.001]. The FamH plus ≥2 healthy lifestyle group had similar age at diagnosis [48.2 (48.1–48.3)] as the non-FamH plus <2 healthy lifestyle group [50.1 (49.8–50.5)]. The FamH group with affected parents had higher odds of hyperglycemia, hypertension, and dyslipidemia than the FamH group with affected siblings, with the lowest odds in the non-FamH group. Self-management (healthy lifestyles plus SMBG) was associated with higher odds of attaining HbA_1c_<7%, blood pressure<130/80mmHg, and LDL-C<2.6 mmol/L especially in the FamH group (FamH×self-management, p_interaction_=0.050–0.001).

**Conclusions:**

In Asia, FamH was common and associated with young age of diagnosis which might be delayed by healthy lifestyle while self management  was associated with better control of  cardiometabolic risk factors especially in those with FamH.

**Supplementary Information:**

The online version contains supplementary material available at 10.1186/s12916-022-02424-y.

## Background

In 2019, 9.3% of the global adult population were affected by diabetes with 50% living in Asia [1]. The majority had type 2 diabetes (95%) characterized by varying degrees of insulin resistance and deficiency [2], often accompanied by clustering of cardiometabolic risk factors [3]. Delayed diagnosis and management of diabetes can lead to poor quality of life, multimorbidity, and premature mortality [4, 5]. Young-onset type 2 diabetes diagnosed before age of 40 has become increasingly prevalent [6]. The growing burden of young-onset type 2 diabetes is multifactorial, including but not limited to genetics, perinatal factors, childhood obesity, and unhealthy lifestyles [6, 7]. In the clinic-based Joint Asia Diabetes Evaluation (JADE) Register, 1 in 5 Asian adults had young-onset type 2 diabetes [8], who had worse control of cardiometabolic risk factors than their peers with late-onset disease [6, 9]. In these young people, decades of exposure to cardiometabolic risk factors can lead to premature complications and death with socioeconomic implications [10].

Family history (FamH) is a strong risk factor for type 2 diabetes [11], evidenced by a higher concordance rate among monozygotic than dizygotic twins [12, 13]. People with FamH of diabetes had early onset of type 2 diabetes [14–18] and increased risks for hypertension, dyslipidemia, and obesity [19, 20]. Apart from shared genotypes, a FamH of diabetes might reflect shared behaviors and environment, such as lifestyles (physical inactivity, unhealthy diet, alcohol, and tobacco use) and socioeconomic status (education, employment, and household income) [20, 21]. On the other hand, some researchers had reported 40–80% reduced risk of type 2 diabetes associated with FamH, probably due to increased perceived risk and motivation to change lifestyles for mitigating risk [22].

Diabetes is a complex disease comprising of, but not limited to, genetic, perinatal, demographic, cognitive-psychosocial-behavioral, environmental, and ecological components with FamH being a proxy of some of these components [2], modified by self-management and access to care to influence age of diagnosis and clinical outcomes (Additional file 1: Figure S1) [2]. Despite this plausibility, there is a paucity of data on the interactive effects between FamH and behavioral factors on disease onset and cardiometabolic risks in type 2 diabetes. In this study, we hypothesize that FamH interacts with unhealthy lifestyles to bring forward age of diagnosis and together with suboptimal self-management, proxied by self-monitoring of blood glucose (SMBG), worsen control of cardiometabolic risk factors compared to those without FamH. We argue that while FamH per se is a non-modifiable risk factor, FamH can be used as a simple proxy to identify high-risk individuals for intensive lifestyle modification to delay disease onset and self-management support programs to improve control of cardiometabolic risk factors. We tested this hypothesis using data from a multicenter diabetes register in Asia with documentation of age of diagnosis, lifestyles, SMBG, and cardiometabolic risk factors.

## Methods

### Study design, setting, and participants

In this cross-sectional analysis, we curated data from the web-based, multi-country, prospective JADE Register, established as part of a quality improvement program to improve care and promote collaborative research in Asia [23]. The JADE Technology was established in 2007 by the Asia Diabetes Foundation, a non-profit research organization governed by the Chinese University of Hong Kong Foundation. The JADE portal adopted the same database structure of the Hong Kong Diabetes Register to guide structured data collection during a clinic visit for risk stratification and promotion of  personalized care. All participating sites were given an operating manual together with the case report form downloadable from the JADE portal which consists of details of rationale, purpose, and protocol including definitions and procedures of the JADE Program [23]. Patients with diabetes were recruited from 427 hospital- and community-based clinics in 11 Asian countries/regions (China, Hong Kong, India, Indonesia, Korea, Malaysia, Philippines, Singapore, Taiwan, Thailand, and Vietnam, refer to Table 1 in Additional file 1). 

The present study included patients who were (1) aged ≥18 years, (2) diagnosed with type 2 diabetes, and (3) enrolled between 2007 and 2021. All patients enrolled in the JADE Register had physician-diagnosed diabetes based on the American Diabetes Association criteria [24] and received routine care at the clinics. At the enrolment visit, patients attended the clinics after at least 8 h of fasting and underwent a structured interview by trained nurses using case report form with pre-defined fields and coded responses. This was followed by collection of fasting blood and early morning urine samples for laboratory assays as well as eye and feet examination according to the standardized JADE protocol. Patients with type 1 diabetes (defined as history of diabetic ketoacidosis and/or requirement of continuous insulin within one year of diagnosis) and unclassified diabetes were excluded [23, 25].

### Exposures and covariates

Collected data included demographics (age and gender), age at diagnosis, types of diabetes, FamH of diabetes affecting first-degree relatives (father, mother, and siblings), education (primary school or below *versus* middle school or above), and employment (non-worker *versus* worker). FamH of diabetes in offsprings was not recorded. Use of medications (yes/no) including oral glucose-lowering drugs, insulin, lipid-lowering drugs, and blood pressure (BP)-lowering drugs was also recorded at enrolment.

Self-reported lifestyle factors and regular SMBG were based on recall in the past 3 months using coded responses. We recorded the frequency of physical activity more than 30 min (none, <3 times, 3–4 times, 5 times, or >5 times weekly), adherence to a balanced  diet (yes, occasionally, or no), use of tobacco (never, ex-, or current smoker), and alcohol (never, ex-, occasional and regular drinker). Healthy lifestyles included adequate physical activity (30 min daily at least thrice weekly), adherence to a balanced  diet (yes), never or ex-smoker, and never or occasional drinker. Self-management was based on the aforementioned 4 lifestyle factors and SMBG (yes/no) and was defined by ≥3 of 5 favorable factors

### Outcomes

The outcomes included age at diagnosis and cardiometabolic risk factors. Overnight fasting blood samples were collected for measuring fasting plasma glucose (FPG), glycated hemoglobin (HbA_1c_), and lipid profile (total cholesterol, high-density lipoprotein cholesterol [HDL-C], calculated low-density lipoprotein cholesterol [LDL-C], and triglyceride). Cardiometabolic risks were defined as follows: hyperglycemia=HbA_1c_>7% (53 mmol/mol) or FPG>7 mmol/L; hypertension=BP≥140/90 mmHg and/or treatment with any BP-lowering drugs; dyslipidemia=LDL-C≥2.6 mmol/L, HDL-C<1.0 mmol/L in men or <1.3 mmol/L in women, triglycerides≥2.3 mmol/L and/or treatment with any lipid-lowering drugs. Optimal control was defined as attainment of ≥2 “ABC” targets (HbA_1c_<7%, BP<130/80 mmHg, LDL-C<2.6 mmol/L) [26, 27] which was associated with 30% reduction in incident cardiovascular disease in Chinese patients with type 2 diabetes [28].

### Data analysis

Patients with complete FamH information (father, mother, and siblings) were included in the analysis. Descriptive statistics were presented as mean±SD or number (percentages) as appropriate. For between-group comparisons, we used the chi-square tests for categorical variables and the Independent *t*-tests for continuous variables. *P*-values adjusted for age, sex, and diabetes duration were further computed using regression methods.

In this cross-sectional analysis of patients with diagnosed diabetes, age at  diagnosis (outcome) and effect of FamH (exposure) occurred before the data collection time-point. As reported by other workers [14], we used Kaplan-Meier estimation to compare the incidence of diabetes (*y*-axis) at each observed event time, i.e., age (*x*-axis) with log-rank *p*-value to provide a visual summary across all time-points. We stratified patients into four groups: (1) non-FamH group with <2 healthy lifestyles, (2) non-FamH group with ≥2 healthy lifestyles, (3) FamH group with <2 healthy lifestyles, and (4) FamH group with ≥2 healthy lifestyles and compared the mean age at diagnosis of type 2 diabetes with 95% confidence interval (95% CI). We performed sensitivity analysis by repeating the Kaplan-Meier analysis on patients diagnosed for less than 1 year prior to baseline assessment to minimize impact of care on behavioral changes.

We conducted binary logistic regression to examine the associations of FamH with cardiometabolic profiles and attainment of “ABC” targets as dependent variables, adjusted for countries/regions, year of enrolment, demographics, socioeconomic status (education and employment), self-management, medication use, and duration of diabetes. We tested for interaction effects between FamH and self-management (lifestyle factors plus SMBG). Given that the interaction terms were not straightforward for interpretation by general readers, we conducted subgroup analysis to demonstrate whether the strength of associations of self-management with dependent variables varied between FamH and non-FamH groups, expressed as adjusted odds ratios (aOR) with 95%CI. We also performed stratified analyses to explore the consistency of the associations across countries/regions. All analyses were conducted using Statistical Package for Social Sciences (IBM, version 26) and R (version 4.0.2). A two-sided P value <0.05 was considered significant. Given the large sample size in the present study, listwise deletion was adopted for handling missing data.

## Results

**Fig. 1:**
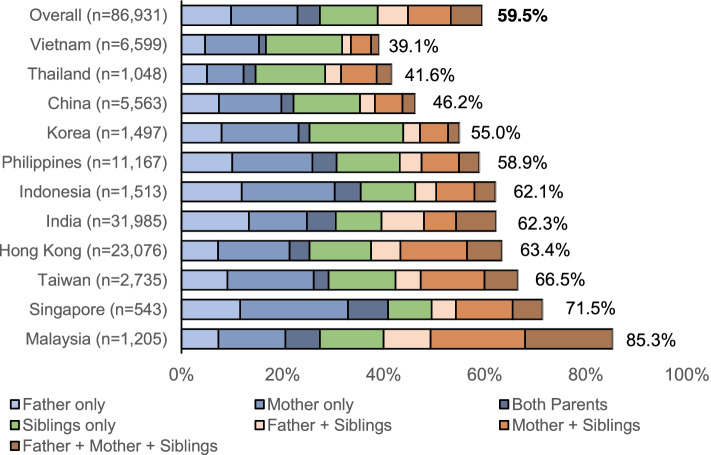
Prevalence of family history among patients with type 2 diabetes across 11 Asian countries/regions. Figures at the end of the bars show the proportions of patients having family history of diabetes (father, mother, and/or siblings) in the corresponding countries/regions

A total of 113,184 patients with diabetes were enrolled in the JADE Register from 2007 to 2021. After excluding patients with type 1 diabetes (*n*=1567) or unclassified diabetes (*n*=8310) and incomplete FamH data (*n*=16,376), we included 86,931 (76.8%) patients with type 2 diabetes [mean±SD age: 56.6±11.6 years, 53.5% men] in our final analysis (Table 1). Overall, 59.5% of the patients reported a positive FamH, with mother-only FamH being most common (13.2%), followed by siblings-only FamH (11.5%) and father-only FamH (9.8%). Among the 11 countries/regions, the prevalence of FamH ranged from 39.1 to 85.3% (Fig. 1). Compared with the non-FamH group, the FamH group was younger without sex preponderance (Table 1). They had better education (middle school or above) and were more likely to be actively employed and perform regular SMBG, but less likely to report a healthy lifestyle (healthy diet, never or ex-smoker, never or occasional alcohol drinker) (adjusted *p*-values<0.05).

**Table 1:** Profile of 86,931 patients with type 2 diabetes in the JADE Register between 2007 and 2021

	Missing	Overall (*n* = 86,931)	Family history (*n* = 51,705)	No family history (*n* = 35,226)	Crude *p*-value	Adjusted *p*-value^#^
*Country/region of recruitment*						
India	0	31,985 (36.8)	19,911 (38.5)	12,074 (34.3)	<0.001	<0.001
Hong Kong	0	23,076 (26.5)	14,632 (28.3)	8444 (24.0)		
Philippines	0	11,167 (12.8)	6578 (12.7)	4589 (13.0)		
Vietnam	0	6599 (7.6)	2577 (5.0)	4022 (11.4)		
China	0	5563 (6.4)	2572 (5.0)	2991 (8.5)		
Taiwan	0	2735 (3.1)	1820 (3.5)	915 (2.6)		
Indonesia	0	1513 (1.7)	940 (1.8)	573 (1.6)		
Korea	0	1497 (1.7)	823 (1.6)	674 (1.9)		
Malaysia	0	1205 (1.4)	1028 (2.0)	177 (0.5)		
Thailand	0	1048 (1.2)	436 (0.8)	612 (1.7)		
Singapore	0	543 (0.6)	388 (0.8)	155 (0.4)		
*Sociodemographic profile*						
Age (years)	60	56.6 ± 11.6	55.2 ± 11.0	58.5 ± 12.0	<0.001	<0.001
Age at diagnosis of diabetes (years)	6285	49.8 ± 10.5	47.9 ± 9.7	52.5 ± 10.9	<0.001	<0.001
Diabetes duration (years)	6282	8.18 ± 7.48	8.74 ± 7.66	7.35 ± 7.12	<0.001	0.011
Sex—male	6	46,487 (53.5)	27,600 (53.4)	18,887 (53.6)	0.480	<0.001
Education						
Primary school or below	2502	18,772 (22.2)	9066 (18.0)	9706 (28.4)	<0.001	<0.001
Middle school and above		65,657 (77.8)	41,233 (82.0)	24,424 (71.6)		
Employment status						
Non-worker	769	47,388 (55.0)	26,663 (51.9)	20,725 (59.6)	<0.001	<0.001
Worker		38,774 (45.0)	24,745 (48.1)	14,029 (40.4)		
Family history						
No family history	0	35,226 (40.5)	-	-	-	-
Father only	0	8534 (9.8)	-	-	-	-
Mother only	0	11,432 (13.2)	-	-	-	-
Both parents	0	3841 (4.4)	-	-	-	-
Siblings only	0	9975 (11.5)	-	-	-	-
Father + siblings	0	5200 (6.0)	-	-	-	-
Mother + siblings	0	7379 (8.5)	-	-	-	-
Father + mothers + siblings	0	5344 (6.1)	-	-	-	-
*Self-management (based on recall in last 3 months)*						
Physical activity (30-min duration)	2855					
<3 times per week		46,445 (55.2)	27,876 (55.4)	18,569 (55.0)	0.264	<0.001
≥3 times per week		37,631 (44.8)	22,443 (44.6)	15,188 (45.0)		
Adherence to a balanced diet	3122					
Never or occasional		40,577 (48.4)	25,105 (50.0)	15,472 (46.1)	<0.001	<0.001
Always		43,232 (51.6)	25,111 (50.0)	18,121 (53.9)		
Use of tobacco	1027					
Never/Ex-smoker		75,500 (87.9)	44,633 (87.0)	30,867 (89.2)	<0.001	<0.001
Current smoker		10,404 (12.1)	6673 (13.0)	3731 (10.8)		
Use of alcohol	1159					<0.001
Never/occasional drinker		65,749 (76.7)	38,414 (75.0)	27,335 (79.2)	<0.001	
Ex-/regular drinker		20,023 (23.3)	12,835 (25.0)	7188 (20.8)		
Self-monitoring of blood glucose	8231	55,472 (70.5)	34,715 (73.5)	20,757 (65.9)	<0.001	<0.001
*Biochemistry*						
HbA1c (%)	10,901	8.00 ± 1.88	8.00 ± 1.82	8.00 ± 1.96	0.839	<0.001
HbA1c (mmol/mol)	10,901	64.0 ± 20.6	64.0 ± 19.9	64.0 ± 21.4	0.839	<0.001
Fasting plasma glucose (mmol/L)	9636	8.39 ± 3.18	8.45 ± 3.11	8.30 ± 3.28	<0.001	0.821
Total cholesterol (mmol/L)	15,389	4.70 ± 1.16	4.68 ± 1.15	4.74 ± 1.19	<0.001	<0.001
HDL-C (mmol/L)	13,426	1.21 ± 0.49	1.20 ± 0.41	1.24 ± 0.59	<0.001	<0.001
LDL-C (mmol/L)	13,699	2.70 ± 1.15	2.68 ± 1.12	2.72 ± 1.20	<0.001	0.660
Triglyceride (mmol/L)	12,472	1.91 ± 2.36	1.88 ± 2.41	1.95 ± 2.30	<0.001	0.151
eGFR (ml/min/1.73m^2^)	15,643	82.0 ± 23.7	83.1 ± 23.6	80.5 ± 23.7	<0.001	0.307
Urinary ACR (mg/mmol)	30,797	20.3 ± 82.0	20.0 ± 80.4	20.8 ± 84.1	0.286	0.001
*Cardiometabolic risk factors*						
Systolic blood pressure (mmHg)	1439	131.0 ± 17.1	131.0 ± 16.9	131.0 ± 17.4	0.758	0.875
Diastolic blood pressure (mmHg)	1599	78.9 ± 9.5	79.0 ± 9.5	78.7 ± 9.6	<0.001	0.003
Body Mass Index (kg/m^2^)	4005	26.2 ± 4.55	26.4 ± 4.50	26.0 ± 4.59	<0.001	0.197
Hyperglycemia^a^	7567	63,397 (79.9)	38,414 (81.1)	24,983 (78.1)	<0.001	0.025
Hypertension^b^	1205	55,618 (64.9)	33,469 (65.5)	22,149 (63.9)	<0.001	<0.001
Dyslipidemia^c^	9417	68,161 (87.9)	40,991 (88.8)	27,170 (86.7)	<0.001	<0.001
*“ABC” treatment goals*^d^						
“A” goal achieved	10,901	25,810 (33.9)	14,718 (32.9)	11,092 (35.4)	<0.001	0.009
“B” goal achieved	1573	24,264 (28.4)	14,633 (28.8)	9631 (27.9)	0.002	<0.001
“C” goal achieved	13,699	37,477 (51.2)	22,323 (51.4)	15,154 (50.8)	0.101	0.532
≥ 2 “ABC” goals achieved	18,005	23,062 (33.5)	13,497 (33.4)	9565 (33.5)	0.672	<0.001
*Drug use at baseline*						
Oral glucose-lowering drug	0	72,318 (83.2)	43,970 (85.0)	28,348 (80.5)	<0.001	<0.001
Insulin	0	21,302 (24.5)	13,322 (25.8)	7980 (22.7)	<0.001	<0.001
Lipid-regulating drug	1197	41,985 (49.0)	26,445 (51.6)	15,540 (45.0)	<0.001	<0.001
Blood pressure-lowering drug	740	47,003 (54.5)	28,756 (55.9)	18,247 (52.5)	<0.001	<0.001
Renin-angiotensin system inhibitors	0	29,199 (33.6)	17,926 (34.7)	11,273 (32.0)	<0.001	<0.001

Family history defined by diabetes affecting father, mother, and/or siblings

*JADE* Joint Asia Diabetes Evaluation, *HbA1c* glycated haemoglobin, *eGFR* estimated glomerular filtration rate, *ACR* albumin-creatinine ratio

Data were expressed as mean ± SD or number (%)

^a^Hyperglycemia = HbA1c > 7% (53 mmol/mol) or fasting plasma glucose > 7 mmol/L

^b^Hypertension = blood pressure ≥ 140/90 mmHg or on any blood pressure-lowering drugs

^c^Dyslipidemia= LDL-C ≥ 2.6 mmol/L, HDL-C < 1 mmol/L, triglycerides ≥ 2.3 mmol/L, or on any lipid-regulating drugs

^d^“ABC” goals refer to HbA1c <7% (53 mmol/mol) (A), blood pressure <130/80 mmHg (B), and LDL-C <2.6 mmol/L (C)

^#^*P*-value adjusted for age, sex and diabetes duration

**Fig. 2:**
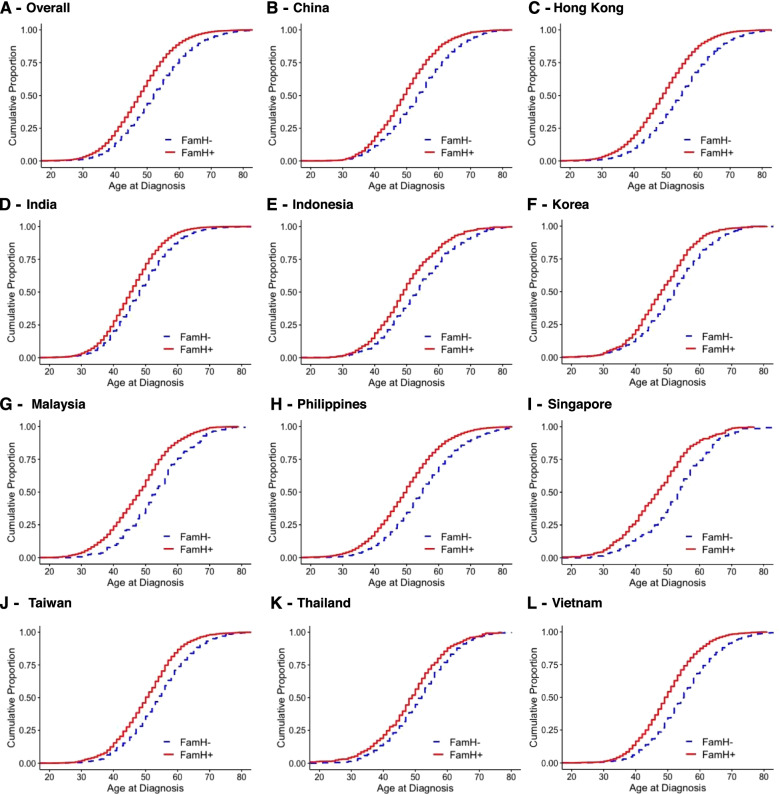
Kaplan-Meier estimate of age at diagnosis stratified by presence of family history. FamH, family history of diabetes (father, mother, and/or siblings). The figure shows the mean age at diagnosis (years) in non-FamH group *vs.* FamH group in (**A**) overall study population [52.5, 95%CI 52.4–52.6] *vs.* [47.9, 95%CI 47.8–49.0], (**B**) China [53.7, 95%CI 53.3–54.1] *vs*. [49.6, 95%CI 49.2–50.0], (**C**) Hong Kong [54.6, 95%CI 54.3–54.8] *vs*. [49.0, 95%CI 48.8–49.2], (**D**) India [48.6, 95%CI 48.4–48.7] *vs.* [45.7, 95%CI 45.6–45.8], (**E**) Indonesia [53.6, 95%CI 52.7–54.6] *vs.* [49.8, 95%CI 49.2–50.5], (**F**) Korea [51.9, 95%CI 51.1–52.7] *vs.* [48.2, 95%CI 47.5–48.9], (**G**) Malaysia [53.4, 95%CI 51.9–55.0] *vs.* [47.9, 95%CI 47.3–48.6], (**H**) Philippines [55.3, 95%CI 54.9–55.6] *vs.* [49.7, 95%CI 49.4–50.0], (**I**) Singapore [53.1, 95%CI 51.3–54.8] *vs.* [46.9, 95%CI 45.7–48.0], (**J**) Taiwan [54.3, 95%CI 53.6–54.9] *vs.* [50.3, 95%CI 49.9–50.8], (**K**) Thailand [51.5, 95%CI 50.6–52.3] *vs.* [48.6, 95%CI 47.5–49.6], and (**L)** Vietnam [54.9, 95%CI 54.6–55.3] *vs.* [49.8, 95%CI 49.4–50.2]. All comparisons showed *p*<0.001 with log-rank test

**Fig. 3:**
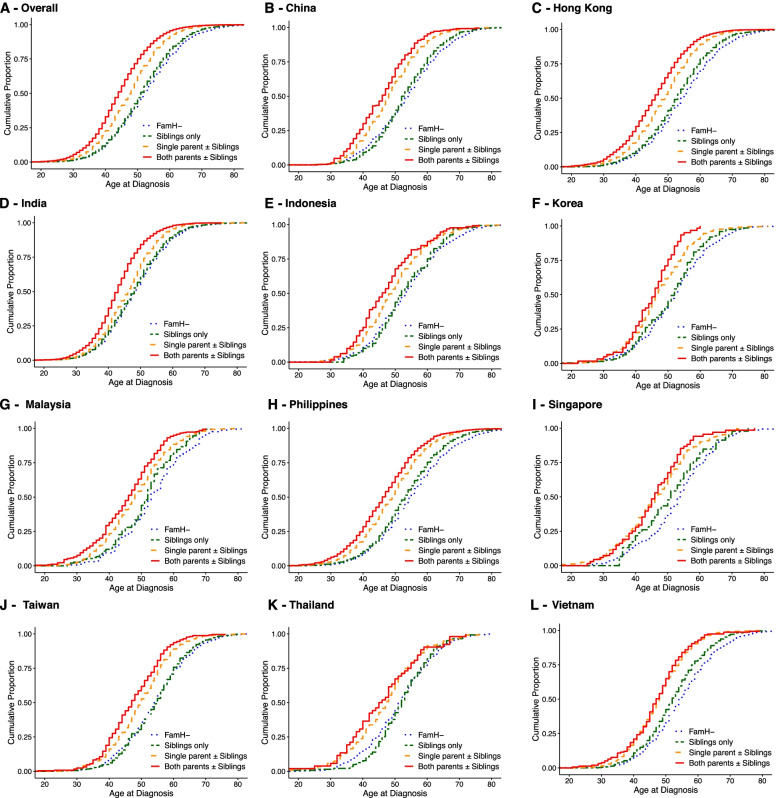
Kaplan-Meier estimate of age at diagnosis stratified by family history affecting parents and/or siblings. FamH, family history of diabetes (father, mother, and/or siblings). The figure shows the mean age at diagnosis (years) in non-FamH group *vs.* FamH group with affected siblings only *vs. *FamH group with affected single parent ± affected siblings *vs.* FamH group with affected both parents ± affected siblings in (**A**) overall study population [52.5, 95%CI 52.4–52.6] *vs.* [51.5, 95%CI 51.3–51.7] *vs.* [47.7, 95%CI 47.6–47.8] *vs.* [44.6, 95%CI 44.5–44.8], (**B**) China [53.7, 95%CI 53.3–54.1] *vs.* [53.3, 95%CI 52.6–54.0] *vs.* [48.4, 95%CI 47.9–48.8] *vs.* [46.1, 95%CI 45.0–47.2], (**C**) Hong Kong [54.6, 95%CI 54.3–54.8] *vs.* [52.8, 95%CI 52.4–53.2] *vs.* [48.7, 95%CI 48.5–48.9] *vs.* [45.7, 95%CI 45.3–46.1], (**D**) India [48.6, 95%CI 48.4–48.7] *vs.* [48.0, 95%CI 47.7–48.4] *vs.* [46.0, 95%CI 45.9–46.2] *vs.* [43.0, 95%CI 42.7–43.2], (**E**) Indonesia [53.6, 95%CI 52.7–54.6] *vs.* [53.2, 95%CI 51.7–54.6] *vs.* [49.5, 95%CI 48.6–50.3] *vs.* [47.4, 95%CI 45.7–49.1], (**F**) Korea [51.9, 95%CI 51.1–52.7] vs. [50.7, 95%CI 49.6–51.9] vs. [47.2, 95%CI 46.3–48.0] *vs.* [44.9, 95%CI 43.0–46.8], (**G**) Malaysia [53.4, 95%CI 51.9–55.0] *vs.* [51.2, 95%CI 49.6–52.7] *vs.* [48.2, 95%CI 47.4–49.1] *vs.* [45.4, 95%CI 44.2–46.7], (**H**) Philippines [55.3, 95%CI 54.9–55.6] *vs.* [53.5, 95%CI 52.9–54.1] *vs.* [49.0, 95%CI 48.7–49.3] *vs.* [46.7, 95%CI 46.0–47.4], (**I**) Singapore [53.1, 95%CI 51.3–54.8] *vs.* [50.9, 95%CI 47.9–54.0] *vs.* [46.4, 95%CI 45.0–47.8] vs. [45.8, 95%CI 43.4–48.1], (**J**) Taiwan [54.3, 95%CI 53.6–54.9] *vs.* [54.2, 95%CI 53.3–55.2] *vs.* [49.7, 95%CI 49.2–50.3] *vs.* [47.5, 95%CI 46.4–48.6], (**K)** Thailand [51.5, 95%CI 50.6–52.3] *vs.* [52.1, 95%CI 50.5–53.6] *vs.* [47.0, 95%CI 45.5–48.4] *vs.* [45.9, 95%CI 42.8–49.1], and (**L**) Vietnam [54.9, 95%CI 54.6–55.3] *vs.* [52.5, 95%CI 51.9–53.1] *vs.* [48.0, 95%CI 47.5–48.5] *vs.* [47.3, 95%CI 45.9–48.8]. All comparisons showed *p* <0.001 with log-rank test

Figures 2 and 3 show the cumulative proportion defined by age at diagnosis across FamH groups. Overall, the FamH group was diagnosed 4.6 years earlier than the non-FamH group [mean (95% CI): 47.9 (47.8–48.0 years) *vs.* 52.5 (52.4–52.6) years] (Fig. 2). Patients with FamH affecting both parents with/without siblings had the earliest age at diagnosis [44.6 (44.5-44.8 years)], followed by FamH affecting single parent with/without siblings [47.7 (47.6-47.8 years)] and FamH affecting siblings only [51.5 (51.3-51.7 years)], with non-FamH group having the oldest age at diagnosis [52.5 (52.4-52.6 years)] (Fig. 3). The findings were consistent across the 11 countries/regions.

**Fig. 4:**
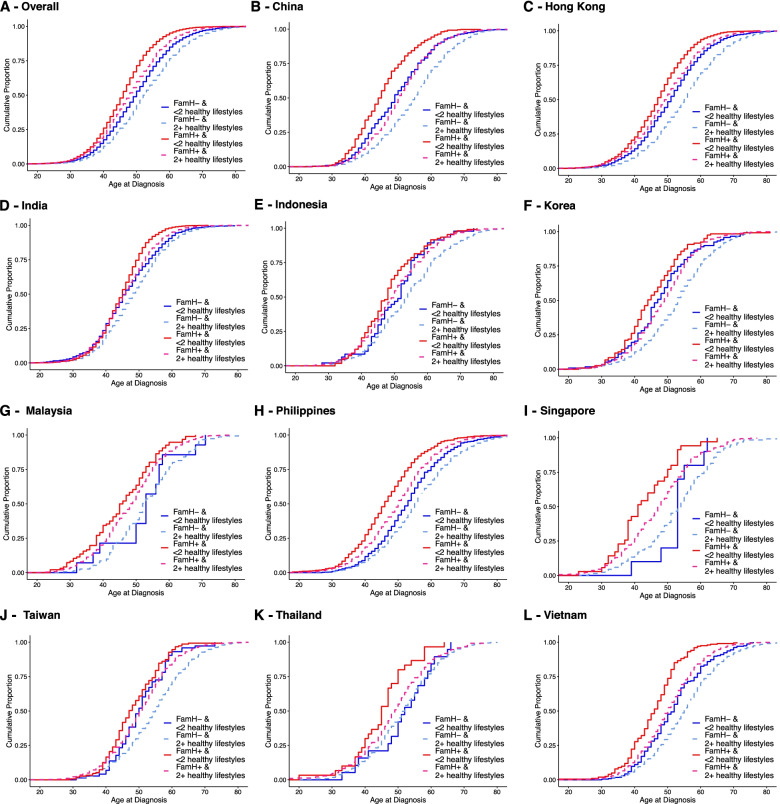
Kaplan-Meier estimate of age at diagnosis stratified by combination of family history and healthy lifestyles. FamH, family history of diabetes (father, mother, and/or siblings). Healthy lifestyles include adequate physical activity (30 min at least 3 times weekly), adherence to a balanced diet, never or occasional alcohol drinker, and never or ex-smoker. The figure shows the mean age at diagnosis (years) in FamH- group with <2 healthy lifestyles *vs.* FamH− group with ≥2 healthy lifestyles *vs.* FamH+ group with <2 healthy lifestyles *vs.* FamH+ group with ≥2 healthy lifestyles in (**A**) overall study population [50.1, 95%CI 49.8–50.5] *vs.* [52.8, 95%CI 52.7–52.9] *vs.* [46.0, 95%CI 45.8–46.2] *vs.* [48.2, 95%CI 48.1–48.3], (**B**) China [53.0, 95%CI 51.5–54.5] *vs.* [57.4, 95%CI 56.3–58.4] *vs.* [50.2, 95%CI 48.6–51.8] vs. [54.3, 95%CI 53.1–55.4], (**C**) Hong Kong [56.0, 95%CI 54.4–57.6] vs. [59.5, 95%CI 58.7–60.2] vs. [52.0, 95%CI 51.1–53.0] vs. [54.5, 95%CI 54.0–55.0], (**D**) India [51.5, 95%CI 49.5–53.5] *vs*. [52.9, 95%CI 52.3–53.5] *vs.* [46.0, 95%CI 45.2–46.9] *vs.* [48.5, 95%CI 48.0–48.9], (**E**) Indonesia [52.8, 95%CI 47.9–57.6] *vs.* [58.8, 95%CI 56.6–60.9] *vs.* [50.2, 95%CI 45.6–54.8] *vs.* [58.0, 95%CI 55.5–60.4], (**F**) Korea [50.9, 95%CI 45.7–56.1] *vs.* [55.3, 95%CI 51.6–59.0] *vs.* [50.3, 95%CI 45.3–55.2] *vs.* [53.1, 95%CI 50.4–55.7], (**G**) Malaysia [52.4, 95%CI 47.1–57.7] *vs.* [53.2, 95%CI 51.6–54.8] *vs.* [45.7, 95%CI 43.8–47.6] *vs.* [48.2, 95%CI 47.5–48.9], (**H**) Philippines [53.7, 95%CI 51.8–55.6] *vs.* [58.6, 95%CI 57.7–59.5] *vs.* [51.6, 95%CI 49.9–53.2] *vs.* [53.7, 95%CI 52.9-54.5], (**I**) Singapore [53.0, 95%CI 53.0–53.0] *vs.* [59.4, 95%CI 54.4–64.4] *vs.* [53.3, 95%CI 49.2–57.3] *vs.* [55.2, 95%CI 52.5-57.9], (**J**) Taiwan [53, 95%CI 53–53] *vs.* [59.4, 95%CI 54.4–64.4] *vs.* [53.3, 95%CI 49.2–57.3] *vs.* [55.2, 95%CI 52.5-57.9], (**K**) Thailand [58.1, 95%CI 55.0–61.3] *vs.* [45.0, 95%CI 40.4–49.6] *vs.* [59.8, 95%CI 53.4–66.2] *vs.* [57.2, 95%CI 54.3–60.0], and (**L**) Vietnam [54.5, 95%CI 51.7–57.4] *vs.* [58.5, 95%CI 57.3–59.6] *vs.* [50.7, 95%CI 47.2–54.2] *vs.* [52.8, 95%CI 51.5–54.1]. All comparisons showed *p* <0.05 with log-rank test

Figure 4 presents the combined associations of lifestyles and FamH with mean age at diagnosis. The non-FamH group with ≥2 healthy lifestyles had the oldest age at diagnosis [52.8 (52.7–52.9) years] while the FamH group with <2 healthy lifestyles had the earliest age at diagnosis [46.0 (45.8–46.2) years]. The FamH group with ≥2 healthy lifestyles had an age at diagnosis [48.2 (48.1–48.3) years] close to the non-FamH group with <2 healthy lifestyles [50.1 (49.8–50.5) years]. Similar patterns were observed in 11 Asian countries/regions. The results of sensitivity analysis (Additional file 1: Figure S2) including patients with type 2 diabetes diagnosed within 1 year before enrolment (*n*=8,556) were consistent with the overall analysis (*n*=86,931).

**Fig. 5:**
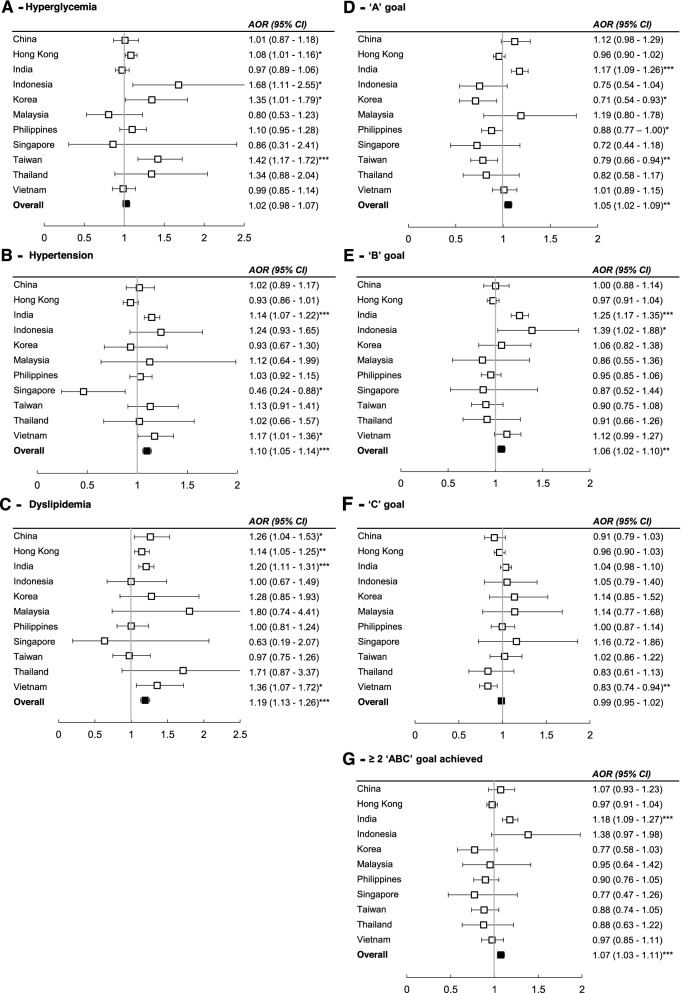
Binary logistic regression of family history of diabetes on cardiometabolic profiles in different countries. All models were adjusted for age, sex, education (middle school and above vs. primary school or below), employment (worker vs. non-worker), drug use (oral glucose-lowering drug, insulin, lipid-regulating drug, blood pressure-lowering drug, and renin-angiotensin system inhibitors), physical activity, adherence to a balanced diet, alcohol use, smoking status, self-monitoring of blood glucose, duration of diabetes, and year of enrolment. The overall association was adjusted for the above variables and country/region of recruitment. Hyperglycemia = HbA1c > 7% (53 mmol/mol) or fasting plasma glucose > 7 mmol/L. Hypertension = blood pressure ≥ 140/90 mmHg or on any blood pressure-lowering drugs. Dyslipidemia= LDL-C ≥ 2.6 mmol/L, HDL-C < 1 mmol/L, triglycerides ≥ 2.3 mmol/L, or on any lipid-regulating drugs. “ABC” goals refer to HbA_1c_ <7% (53 mmol/mol) (**A**), blood pressure <130/80 mmHg (**B**), and LDL-C <2.6 mmol/L (**C**). ***p<0.001, **p<0.01, *p<0.05; AOR = Adjusted odds ratios, 95% CI = 95% confidence interval

**Fig. 6:**
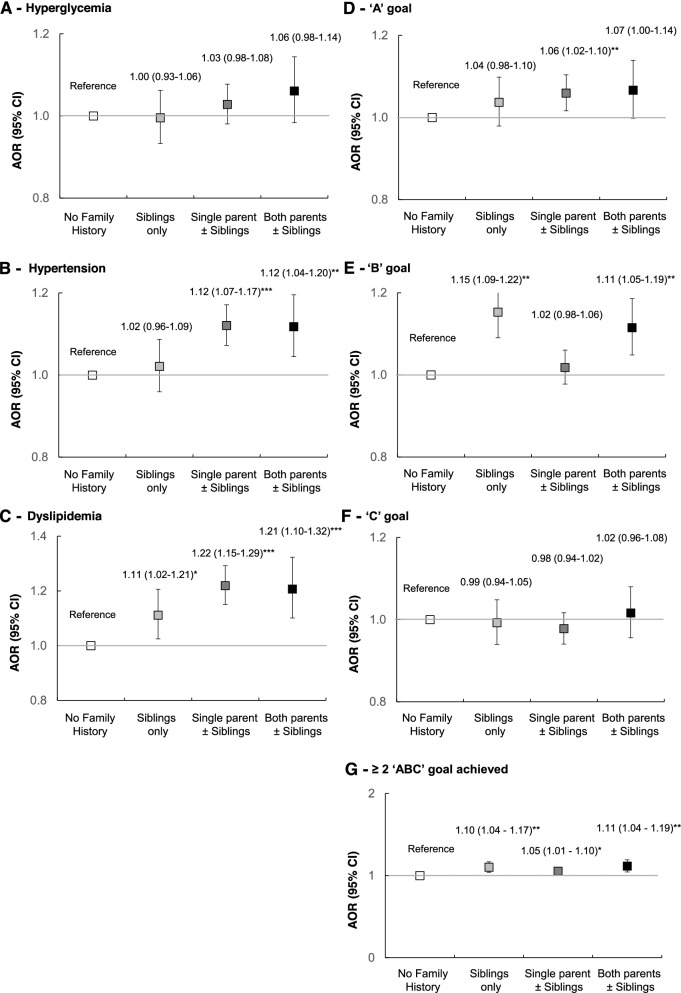
Binary logistic regression of family history affecting parents and/or siblings on cardiometabolic profiles. All models were adjusted for age, sex, education (middle school and above *vs.* primary school or below), employment (worker *vs.* non-worker), drug use (oral glucose-lowering drug, insulin, lipid regulating drug, blood pressure-lowering drug, and renin-angiotensin system inhibitors), physical activity, adherence to a balanced diet, alcohol use, smoking status, self-monitoring of blood glucose, duration of diabetes, year of enrolment, and country/region of recruitment. Hyperglycemia = HbA_1c_ > 7% (53 mmol/mol) or fasting plasma glucose > 7 mmol/L. Hypertension = blood pressure ≥ 140/90 mmHg or on any blood pressure-lowering drugs. Dyslipidemia= LDL-C ≥ 2.6 mmol/L, HDL-C < 1 mmol/L, triglycerides ≥ 2.3 mmol/L, or on any lipid-regulating drugs. “ABC” goals refer to HbA_1c_ <7% (53 mmol/mol) (**A**), blood pressure <130/80 mmHg (**B**), and LDL-C <2.6 mmol/L (**C**). ***p<0.001, **p<0.01, *p<0.05. AOR = adjusted odds ratios, 95% CI = 95% confidence interval

After adjusting for country, years of registration, demographics, education, employment status, lifestyles, SMBG, and medication use at registration, the FamH group had higher odds of hypertension (aOR 1.10, 95% CI 1.05–1.14) and dyslipidemia (aOR 1.19, 95% CI 1.13–1.26) but also higher odds of achieving the “A” goal (aOR 1.05, 95% CI 1.02–1.09), “B” goal (aOR 1.06, 95% CI 1.02–1.10), and ≥2 “ABC” goals (aOR 1.07, 95% CI 1.03–1.11), albeit with considerable inter-country variations (Fig. 5). Patients with affected parents with/without siblings also had higher adjusted odds of hyperglycemia, hypertension, and dyslipidemia compared to patients with affected siblings only (Fig. 6).

**Fig. 7:**
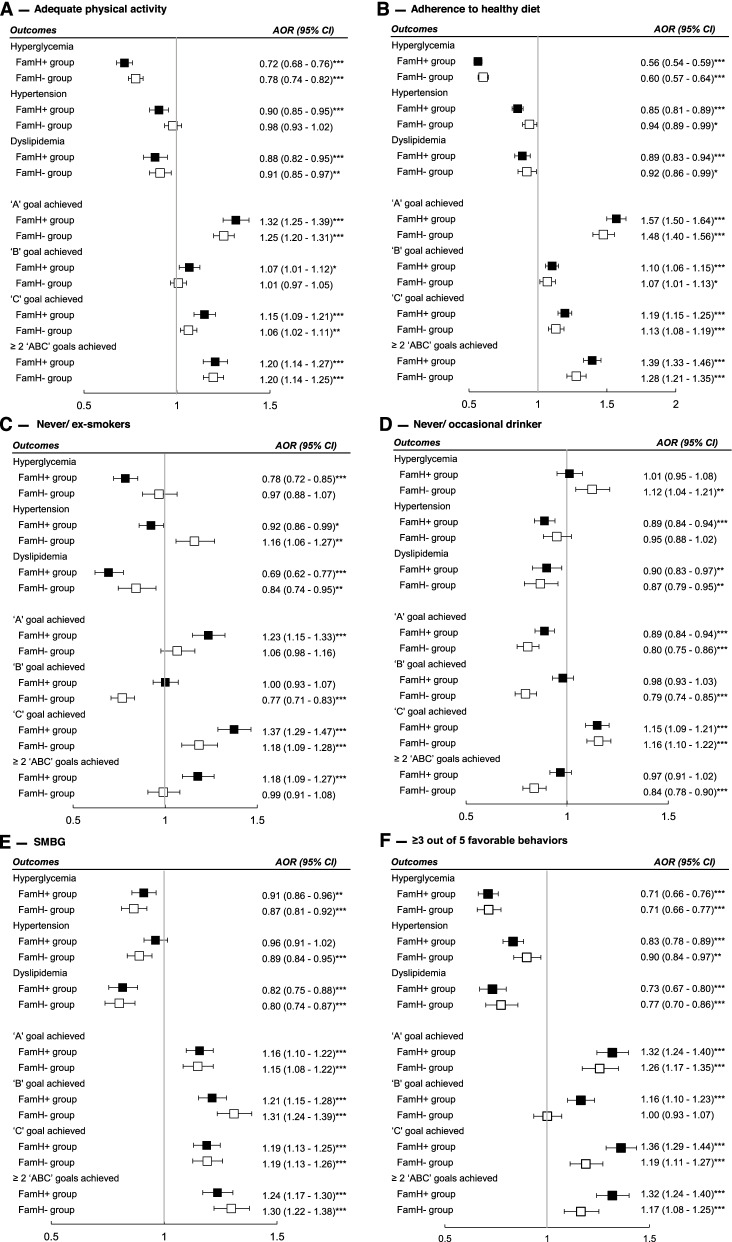
Binary logistic regression of self-management stratified by family history on cardiometabolic profiles. Self-management was defined as ≥3 of 5 behavioral factors including adequate physical activity (30 min at least 3 times weekly), adherence to a balanced diet, never or occasional alcohol drinker, and never or ex-smoker, or self-monitoring of blood glucose. All models were adjusted for age, sex, education (middle school and above *vs.* primary school or below), employment (worker *vs.* non-worker), drug use (oral glucose-lowering drug, insulin, lipid regulating drug, blood pressure-lowering drug, and renin-angiotensin system inhibitors), duration of diabetes, year of enrolment, and country/region of recruitment. Hyperglycemia = HbA_1c_ > 7% (53 mmol/mol) or fasting plasma glucose > 7 mmol/L. Hypertension = blood pressure ≥ 140/90 mmHg or on any blood pressure-lowering drugs. Dyslipidemia= LDL-C ≥ 2.6 mmol/L, HD-CL < 1 mmol/L, triglycerides ≥ 2.3 mmol/L, or on any lipid-regulating drugs. “ABC” goals refer to HbA_1c_ <7% (53 mmol/mol) (**A**), blood pressure <130/80 mmHg (**B**), and LDL-C <2.6 mmol/L (**C**). ***p<0.001, **p<0.01, *p<0.05. FamH = family history of diabetes (father, mother, and/or siblings). AOR, adjusted odds ratios; 95% CI, 95% confidence interval

Self-management (healthy lifestyles plus SMBG) was associated with lower odds of hyperglycemia, hypertension, and dyslipidemia, and higher odds of attaining each “ABC” goal in both FamH and non-FamH groups, with greater strength of associations in the FamH group (Fig. 7F). The interaction term (FamH × self-management) was associated with lower odds of hypertension (aOR 0.90, 95% CI 0.82–0.98) and higher odds of achieving the “A” goal (aOR 1.10, 95% CI 1.00–1.20), “B” goal (aOR 1.20, 95% CI 1.10–1.31), “C” goal (aOR 1.18, 95% CI 1.08–1.28), and ≥ 2 “ABC” goals (aOR 1.19, 95% CI 1.08–1.30) (Additional file 1: Table S2). Subgroup analyses showed interaction between each behavior and FamH (Fig. 7A–D) except for SMBG with similar effect size in FamH and non-FamH group (Additional file 1: Table S2) while never or occasional use of alcohol was associated with lower odds of “A” and “B” goal achievement.

## Discussion

Based on hypothesis defined a priori, to the best of our knowledge, this is the first real-world evidence on the combined associations of  FamH and behavioral factors with  age at  diagnosis and control of cardiometabolic risk factors in Asian patients with type 2 diabetes. In this study, the proportion of patients with FamH of diabetes ranged from 39.1% in Vietnam to 85.3% in Malaysia, similar to the 40–60% prevalence of FamH reported from Korea, India, and Belgium [14–17], Overall, the FamH group was diagnosed 4.6 years younger than the non-FamH group which was the same as that reported in a Korean study [14]. In Sydney, other researchers also showed earlier age of diagnosis by 1.7 years for every 10% increase in affected family members [29].

The FamH plus unhealthy lifestyle group had the earliest age at diagnosis (46.0 years) while the non-FamH plus healthy lifestyle group had the oldest age at diagnosis (52.8 years). Interestingly, the FamH plus healthy lifestyle group (48.2 years) had similar age at diagnosis as the non-FamH plus unhealthy lifestyle group (50.1 years). This trend was found in both newly diagnosed patients and those with established diabetes. In part due to their younger age and active work-life, the FamH group was less likely to report healthy lifestyles with suboptimal control of cardiometabolic risk factors. While self-management (healthy lifestyle plus SMBG) was associated with better control of cardiometabolic risk factors and attainment of treatment goals in both FamH and non-FamH groups, this association was more marked in the FamH group with significant interaction. Although some researchers had specifically reported that healthy lifestyles might delay the onset of diabetes [30], no stratified analysis by FamH was reported.

The proportions of patients with FamH vary amongst different countries/regions. This might reflect different levels of public awareness regarding the familial nature of diabetes and access to early detection programs. Despite differences in national income levels, health systems and access to medicines, care, and support, the interactions between FamH and self-management on age at diagnosis and control of cardiometabolic risk factors were consistent across all participating Asian countries or areas. In our study, 1 in 3 patients reported a maternal history of diabetes with or without other affected family members. This accords with the known risk associations of maternal hyperglycemia with early-onset diabetes in the offspring [2, 31], likely attributable to intrauterine effects of maternal obesity and gestational diabetes [6]. Apart from shared environment, lifestyles, common and rare genetic factors [32], chronic hepatitis B infection, and hemoglobinopathy (affecting 6–10% of the Asian population) with familial clustering were associated with increased risk of diabetes. These risk associations might be due to low grade inflammation and oxidative stress, which might contribute to the familial clustering of diabetes [33, 34].

In our literature search, we did not find direct evidence suggesting that FamH raised awareness resulting in early screening and younger age of diagnosis. Some researchers had reported that African Americans with FamH were more aware of risk factors for diabetes and more likely to consume fruits and vegetables and engage in diabetes screening [35]. This might lead to a younger age of diagnosis, especially in healthcare systems with easy access to screening service. However, in our study, patients with FamH were less likely to report healthy lifestyles, which might interact with genetic factors to bring forward age at  diagnosis. On the other hand, compared with those without FamH, they were more likely to perform SMBG. Given the benefits of peer support on self-management [36], we hypothesize that mutual support among affected family members might motivate increased use of SMBG to control blood glucose. In the International Diabetes Mellitus Practice Survey recruiting patients outside Europe and North America, SMBG was the only factor associated with attainment of HbA_1c_ goal across all regions [27]. Data from the Taiwan Diabetes Registry also shows that SMBG was associated with higher odds of HbA_1c_<7% in patients with recently diagnosed type 2 diabetes [35]. Qualitative analysis through direct interview may provide more insights on differences in behavioral determinants such as values, perspectives, and concerns between people with or without FamH [2].

### Implication

There is a wealth of randomized controlled trials showing that diabetes can be prevented in high-risk individuals, although few studies highlighted the delayed age at  diagnosis [2]. Although some researchers had specifically reported that healthy lifestyles might delay the onset of diabetes [30], no stratified analysis by FamH was reported. Results from our analysis suggested that FamH, especially affecting parents and/or siblings, brought forward the age at  diagnosis by nearly 5 years although this could be delayed by healthy lifestyles. Similarly, although patients with FamH had worse control of cardiometabolic risk factors than the non-FamH group, they appeared to benefit more from self-management (lifestyles plus SMBG).

We leveraged the structured data collection of the JADE Register to explore the associations of FamH with age at diagnosis, cardiometabolic risks, and attainment of treatment targets, which if sustained, would reduce clinical events in the long term [28]. In this light, the JADE Register systematically gathered data in real-world practice to issue a personalized report complete with risk stratification (including FamH), complications, targets/trends of risk profile, and lifestyle factors to promote self-management and early intervention [36].

Self-management is the cornerstone in diabetes prevention and treatment [37]. In this analysis, patients with FamH and healthy lifestyles had an age at  diagnosis close to those without FamH and unhealthy lifestyles. Together with SMBG, these positive health behaviors had greater effect size in achieving treatment targets in patients with FamH than those without. While many experts advocate the use of biogenetic markers and algorithms to improve prediction, diagnosis, and management of patients with complex diseases such as type 2 diabetes [37], our data suggested that FamH is a simple proxy which can be used to identify high-risk individuals for intensive education and empowerment to delay disease onset and improve clinical outcomes.

### Strength, limitations, and future study

The JADE Register enrolled patients from a wide range of hospital and community-based clinics in different countries with universal, subsidized, or private payment structures. This heterogeneity improved the generalizability of our findings across Asia. Our study has several limitations. First, the cross-sectional design precludes elucidation of a causal relationship of behavioral factors with age at diagnosis and cardiometabolic risks. Routine screening in some participating sites with specialized diabetes centers might have led to earlier diagnosis of patients with FamH. On the other hand, in some low- and middle-income countries where people have to pay out of pocket for screening, this might lead to delayed diagnosis. Although there was heterogeneity in health systems across the Asian regions, we had included region and year of enrolment as covariates in the regression models which yielded similar results on country-specific analysis.

Given their exposure to an affected family member, the interaction between FamH and self-management on risk factor control is plausible. The negative associations between healthy lifestyle and age at  diagnosis might be confounded by behavioral changes after diagnosis although results were consistent in newly diagnosed patients. The JADE Register uses a pragmatic design to implement data-driven integrated care in a real-world setting [38, 39]. Thus, instead of using complex instruments, we used simple questionnaires based on available evidence to assess behavioral factors. The self-report of lifestyles and regular SMBG might be subject to social desirability bias although the use of coded responses in the case report form increased the internal validity. Recall bias might also introduce uncertainty of data such as age at diagnosis especially for silent disease such as diabetes. We also did not ascertain the FamH by confirming with the family members.

## Conclusions

A FamH of diabetes is a complex indicator of the multicausality of type 2 diabetes. While FamH and non-white ethnicity are well-known risk factors for type 2 diabetes, for the first time, our real-world evidence had quantified the age at diagnosis in patients with or without FamH and their associations with cardiometabolic risks, with potential modification by self-management. Given the legacy effect of glycemic control on future clinical events [40, 41], early detection of prediabetes in family members of affected patients for lifestyle intervention might delay onset of diabetes. In patients with familial diabetes, promoting self-management might be particularly effective in controlling cardiometabolic risk factors. These results supported the recommendation of implementing a data-driven integrated diabetes program to adopt a family-based approach to detect, prevent, and treat diabetes focusing on empowerment with ongoing support [42].

## Supplementary Information

**Additional file 1: Table S1.** [List of 427 hospital- and community-based clinics in 11 Asian countries/regions included]. **Table S2.** [Interaction effects of family history of diabetes and self-management on cardiometabolic risk factors]. **Figure S1.** [Conceptual framework of the complex interactions between family history and behavioral factors on development of type 2 diabetes and cardio-metabolic control]. **Figure S2.** [Kaplan–Meier estimate of cumulative proportion for age at diagnosis among 8,556 patients with type 2 diabetes diagnosed within 1 year prior to assessment].

## Data Availability

Data cannot be released without patients’ consent in the public domain for open, unrestricted access. Researchers who are interested and meet the criteria for research access to our data may apply via Asia Diabetes Foundation (enquiry@adf.org.hk).

## Abbreviations

BP: Blood pressure
FamH: Family history
FPG: Fasting plasma glucose
HDL-C: High-density lipoprotein cholesterol
JADE: Joint Asia Diabetes Evaluation
LDL-C: Low-density lipoprotein cholesterol
SMBG: Self-monitoring of blood glucose

## References

[1] Saeedi P, Petersohn I, Salpea P, Malanda B, Karuranga S, Unwin N (2019). Global and regional diabetes prevalence estimates for 2019 and projections for 2030 and 2045: Results from the International Diabetes Federation Diabetes Atlas, 9(th) edition. Diabetes Res Clin Pract..

[2] Chan JCN, Lim LL, Wareham NJ, Shaw JE, Orchard TJ, Zhang P (2020). The Lancet Commission on diabetes: using data to transform diabetes care and patient lives. Lancet..

[3] Chan JCN, Yeung R, Luk A (2014). The Asian diabetes phenotypes: challenges and opportunities. Diabetes Res Clin Pract..

[4] Jiang HJ, Stryer D, Friedman B, Andrews R (2003). Multiple hospitalizations for patients with diabetes. Diabetes Care.

[5] Tabish SA (2007). Is diabetes becoming the biggest epidemic of the twenty-first century?. Int J Health Sci (Qassim)..

[6] Luk AOY, Ke C, Lau ESH, Wu H, Goggins W, Ma RCW (2020). Secular trends in incidence of type 1 and type 2 diabetes in Hong Kong: a retrospective cohort study. PLOS Med..

[7] Mayer-Davis EJ, Lawrence JM, Dabelea D, Divers J, Isom S, Dolan L (2017). Incidence trends of type 1 and type 2 diabetes among youths, 2002–2012. N Engl J Med..

[8] Yeung RO, Zhang Y, Luk A, Yang W, Sobrepena L, Yoon K-H (2014). Metabolic profiles and treatment gaps in young-onset type 2 diabetes in Asia (the JADE programme): a cross-sectional study of a prospective cohort. Lancet Diabetes Endocrinol..

[9] Zoungas S, Woodward M, Li Q, Cooper ME, Hamet P, Harrap S (2014). Impact of age, age at diagnosis and duration of diabetes on the risk of macrovascular and microvascular complications and death in type 2 diabetes. Diabetologia..

[10] Ke C, Lau E, Shah BR, Stukel TA, Ma RC, So W-Y (2019). Excess burden of mental illness and hospitalization in young-onset type 2 diabetes. Ann Intern Med..

[11] Papazafiropoulou AK, Papanas N, Melidonis A, Maltezos E (2017). Family history of type 2 diabetes: does having a diabetic parent increase the risk?. Curr Diabetes Rev..

[12] Sanghera DK, Blackett PR (2012). Type 2 diabetes genetics: beyond GWAS. J Diabetes Metab..

[13] Poulsen P, Grunnet LG, Pilgaard K, Storgaard H, Alibegovic A, Sonne MP (2009). Increased risk of type 2 diabetes in elderly twins. Diabetes..

[14] Noh J-W, Jung JH, Park JE, Lee JH, Sim KH, Park J (2018). The relationship between age of onset and risk factors including family history and life style in Korean population with type 2 diabetes mellitus. J Phys Ther Sci..

[15] Jeong SU, Kang DG, Lee DH, Lee KW, Lim D-M, Kim BJ (2010). Clinical characteristics of type 2 diabetes patients according to family history of diabetes. Korean Diabetes J..

[16] Geetha A, Gopalakrishnan S, Umadevi R (2017). Study on the impact of family history of diabetes among type 2 diabetes mellitus patients in an urban area of Kancheepuram district, Tamil Nadu. Int J Community Med Public Heal..

[17] Hermans MP, Ahn SA, Rousseau MF (2019). Crossing family histories of diabetes and cardiovascular disease leads to unexpected outcomes in diabetic offspring. J Diabetes..

[18] Ng MC, Lee SC, Ko GT, Li JK, So WY, Hashim Y (2001). Familial early-onset type 2 diabetes in Chinese patients: obesity and genetics have more significant roles than autoimmunity. Diabetes Care..

[19] Li JKY, Ng MCY, So WY, Chiu CKP, Ozaki R, Tong PCY (2006). Phenotypic and genetic clustering of diabetes and metabolic syndrome in Chinese families with type 2 diabetes mellitus. Diabetes Metab Res Rev..

[20] Wu H, Lau ES, Kong AP, Ma RC, Ozaki R, Cheung KK (2018). Association between educational level and cardiovascular disease and all-cause mortality in patients with type 2 diabetes: a prospective study in the Joint Asia Diabetes Evaluation Program. Clin Epidemiol..

[21] Cho NH, Chan JCN, Jang HC, Lim S, Kim HL, Choi SH (2009). Cigarette smoking is an independent risk factor for type 2 diabetes: a four-year community-based prospective study. Clin Endocrinol (Oxf)..

[22] Choi J, Choi J-Y, Lee S-A, Lee K-M, Shin A, Oh J (2019). Association between family history of diabetes and clusters of adherence to healthy behaviors: cross-sectional results from the Health Examinees-Gem (HEXA-G) study. BMJ Open..

[23] Chan JCN, Lim L-L, Luk AOY, Ozaki R, Kong APS, Ma RCW (2019). From Hong Kong Diabetes Register to JADE Program to RAMP-DM for data-driven actions. Diabetes Care..

[24] American Diabetes Association (2020). 2. Classification and Diagnosis of Diabetes: Standards of Medical Care in Diabetes—2021. Diabetes Care.

[25] Piwernetz K, Home PD, Snorgaard O, Antsiferov M, Staehr-Johansen K, Krans M (1993). Monitoring the targets of the St Vincent Declaration and the implementation of quality management in diabetes care: the DIABCARE initiative. The DIABCARE Monitoring Group of the St Vincent Declaration Steering Committee. Diabet Med..

[26] Davies MJ, D’Alessio DA, Fradkin J, Kernan WN, Mathieu C, Mingrone G (2018). Management of Hyperglycemia in Type 2 Diabetes. A consensus report by the American Diabetes Association (ADA) and the European Association for the Study of Diabetes (EASD). Diabetes Care..

[27] Chan JCN, Gagliardino JJ, Baik SH, Chantelot J-M, Ferreira SRG, Hancu N (2009). Multifaceted determinants for achieving glycemic control: the International Diabetes Management Practice Study (IDMPS). Diabetes Care..

[28] Kong APS, Yang X, Ko GTC, So W-Y, Chan W-B, Ma RCW (2007). Effects of treatment targets on subsequent cardiovascular events in Chinese patients with type 2 diabetes. Diabetes Care..

[29] Molyneaux L, Constantino M, Yue D (2004). Strong family history predicts a younger age of onset for subjects diagnosed with type 2 diabetes. Diabetes Obes Metab..

[30] Mozaffarian D, Kamineni A, Carnethon M, Djoussé L, Mukamal KJ, Siscovick D (2009). Lifestyle risk factors and new-onset diabetes mellitus in older adults: the cardiovascular health study. Arch Intern Med..

[31] Tam WH, Ma RCW, Ozaki R, Li AM, Chan MHM, Yuen LY (2017). In utero exposure to maternal hyperglycemia increases childhood cardiometabolic risk in offspring. Diabetes Care..

[32] McCarthy MI (2010). Genomics, type 2 diabetes, and obesity. N Engl J Med..

[33] Li-Ng M, Tropp S, Danoff A, Bini EJ (2007). Association between chronic hepatitis B virus infection and diabetes among Asian Americans and Pacific Islanders. Dig Liver Dis..

[34] Lao TT, Chan BCP, Leung W-C, Ho L-F, Tse K-Y (2007). Maternal hepatitis B infection and gestational diabetes mellitus. J Hepatol..

[35] Wang J-S, Lo S-H, Yeh Y-P, Hwu C-M, Huang C-N, Hsieh C-H (2021). Distinct associations of self-monitoring of blood glucose with glycemic control and hypoglycemia between groups of recently diagnosed and long-term follow-up type 2 diabetes: the Taiwan Diabetes Registry. Int J Clin Pract..

[36] Chan JCN, Ozaki R, Luk A, Kong APS, Ma RCW, Chow FCC (2014). Delivery of integrated diabetes care using logistics and information technology-the Joint Asia Diabetes Evaluation (JADE) program. Diabetes Res Clin Pract..

[37] Chatterjee S, Davies MJ, Heller S, Speight J, Snoek FJ, Khunti K (2018). Diabetes structured self-management education programmes: a narrative review and current innovations. Lancet Diabetes Endocrinol..

[38] Lim L-L, Lau ESH, Fu AWC, Ray S, Hung Y-J, Tan ATB (2021). Effects of a technology-assisted integrated diabetes care program on cardiometabolic risk factors among patients with type 2 diabetes in the Asia-Pacific region: the JADE Program randomized clinical trial. JAMA Netw Open..

[39] Chan JCN, Thewjitcharoen Y, Nguyen TK, Tan A, Chia Y-C, Hwu C-M (2022). Effect of a web-based management guide on risk factors in patients with type 2 diabetes and diabetic kidney disease: a JADE randomized clinical trial. JAMA Netw Open..

[40] Laiteerapong N, Ham SA, Gao Y, Moffet HH, Liu JY, Huang ES (2019). The legacy effect in type 2 diabetes: impact of early glycemic control on future complications (The Diabetes & Aging Study). Diabetes Care..

[41] Ke C, Stukel TA, Shah BR, Lau E, Ma RC, So W-Y (2020). Age at diagnosis, glycemic trajectories, and responses to oral glucose-lowering drugs in type 2 diabetes in Hong Kong: a population-based observational study. PLoS Med..

[42] Lim LL, Lau ESH, Kong APS, Davies MJ, Levitt NS, Eliasson B (2018). Aspects of multicomponent integrated care promote sustained improvement in surrogate clinical outcomes: a systematic review and meta-analysis. Diabetes Care..

