# Supplementary material for: Combined associations of family history and self-management with age at diagnosis and cardiometabolic risk in 86,931 patients with type 2 diabetes: Joint Asia Diabetes Evaluation (JADE) Register from 11 countries
Source: BMC Med. 2022 Jul 14;20:249. doi: 10.1186/s12916-022-02424-y (PMC9281062; doi:10.1186/s12916-022-02424-y)
Supplement: Supplementary file 1 — Additional file 1: Table S1. [List of 427 hospital- and community-based clinics in 11 Asian countries/regions included]. Table S2. [Interaction effects of family history of diabetes and self-management on cardiometabolic risk factors]. Figure S1. [Conceptual framework of the complex interactions between family history and behavioral factors on development of type 2 diabetes and cardio-metabolic control]. Figure S2. [Kaplan–Meier estimate of cumulative proportion for age at diagnosis among 8,556 patients with type 2 diabetes diagnosed within 1 year prior to assessment]. [file 12916_2022_2424_MOESM1_ESM.docx]

**Additional file**

Table S1. List of 427 hospital- and community-based clinics in 11 Asian countries/regions included

| **Countries/region** | **Clinics** |
| --- | --- |
| China | Peking University 1st Affiliated Hospital |
| China | Peking Union Hospital |
| China | China-Japan Friendship Hospital |
| China | Chinese Medicine Hospital of Anhui Province |
| China | First People's Hospital of Kunshan City |
| China | HuaiAn Second People's Hospital |
| China | Jiangsu Province Official Hospital |
| China | Peking University People’s Hospital |
| China | Rehabilitation Hospital of Zhenjiang, Jiangsu Province |
| China | Second Affiliated Hospital of Suzhou University |
| China | Shanghai Jiaotong University No.6 People Hospital |
| China | The Affiliated Hospital of Jiangsu-Nantong University |
| China | The Second Hospital of Dalian Medical University |
| China | Third Affiliated Hospital of Sun Yat Sen University |
| China | Third People's Hospital of Hefei City |
| Hong Kong | Alice Ho Miu Ling Nethersole Hospital |
| Hong Kong | The Chinese University of Hong Kong |
| Hong Kong | Dr. Kevin Ho Clinic |
| Hong Kong | Health Logistics |
| Hong Kong | Dr. Chiu Kun Ming Clinic |
| Hong Kong | Heartlink – Dr. Tai Chun Chung |
| Hong Kong | Dr. Lam Ngam Clinic |
| Hong Kong | Dr. Leung Kam Fai Clinic |
| Hong Kong | Dr. Leung Tin Ming Clinic |
| Hong Kong | Dr. Li Shing Yan Clinic |
| Hong Kong | Dr. Poon Shiu Hong Clinic |
| Hong Kong | Dr. Tan TP Clinic |
| Hong Kong | Dr. Tuet On Sany Clinic |
| Hong Kong | Dr. Wong King Wai Clinic |
| Hong Kong | Dr. Wong Tsz Kau Clinic |
| Hong Kong | Dr. Wong Yan Bui Clinic |
| Hong Kong | Dr. Yau Chi Hong Clinic |
| Hong Kong | Ma On Shan Family Medicine Centre |
| Hong Kong | North District Hospital |
| Hong Kong | Prince of Wales Hospital – Diabetes and Endocrine Centre |
| Hong Kong | Qualigenics |
| Hong Kong | Raffles Medical Service Hong Kong |
| Hong Kong | University Health Service, Hong Kong Polytechnic University |
| Hong Kong | CUHK Yao Chung Kit Diabetes Assessment Centre |
| India | A.J. Hospital & Research Centre |
| India | A.T. Diabetes Centre |
| India | Aastha Lifecare Hospital |
| India | Priya Nursing Home |
| India | Arthur Asirvatham Hospital |
| India | Agarwal Heart and Diabetes Clinic |
| India | Aggarwal Centre for Diabetes and Cancer Care |
| India | Alpha Hospital and Research Centre |
| India | AMRI Hospital |
| India | Apollo Hospitals New Friends Colony |
| India | Apollo Hospital |
| India | Apollo Hospitals Rabjyot Nest |
| India | Arthur Asirvatham Hospital |
| India | Ashiwini Kidney And Dialysis Centre |
| India | Asian Diabetic Centre |
| India | Ayushyam Clinic |
| India | Bangalore Diabetes Centre |
| India | Basu's Clinic |
| India | Belgaum Diabetes Centre |
| India | BGS Apollo Hospital |
| India | Bliss Clinic |
| India | Care and Cure Clinic |
| India | Care Diabetes Centre |
| India | Centre for Diabetes and Endocrine Care |
| India | Centre for Diabetes and Endocrine Diseases |
| India | Centre for Diabetes Care and Prevention |
| India | Chellam Hospital |
| India | Chetana Clinic |
| India | Chitransh Diabetic Care Clinic |
| India | City Hospital |
| India | Coimbatore Diabetes Foundation Resort Hospital |
| India | Daksha Medical and Research Centre |
| India | Delhi Diabetes Research Centre |
| India | Deogaonkar Hospital |
| India | Dhanvantari Hospital |
| India | Dia Care |
| India | Diab Care India |
| India | Diabetes Care and Research Centre |
| India | Diabetes Care Center |
| India | Diabetes Care Clinic |
| India | Diabetes Clinic and Research Centre |
| India | Diabetes Hospital And Research Centre |
| India | Diabetes Medicare Centre |
| India | Diabetes, Thyroid and Endocrine Center |
| India | Diabetes, Thyroid and Hormones Centre |
| India | Diabetic Care Centre |
| India | Dia Care - Diabetes Care & Hormone Clinic |
| India | Diacare |
| India | Diaclinic |
| India | Diacon Hospital |
| India | Dianova Diabetes Centre |
| India | Dr. Ajay Aggarwal's Clinic |
| India | Dr. Baliarsinha Endocrine Clinic |
| India | Dr. K. Hari’s Clinic |
| India | Dr. Kovil's Diabetes Care Centre |
| India | Dr. Mohan's Diabetes Specialities Centre |
| India | Dr. Mundhra's Clinic |
| India | Dr. Oak Hospital |
| India | Dr. Rasheed's Diabetes Speciality Centre |
| India | Dr. Vinay Dhandhania's Diabetes Care Clinic |
| India | Dr. Amit Roy's Clinic |
| India | Dr. Aravind's Diabetes Centre |
| India | Dr. Ashok Sharma's Clinic |
| India | Dr. Ghoshdastidar's Clinic |
| India | Dr. Goswami's Clinic |
| India | Dr. Goyal's Clinic |
| India | Dr. J. Murali's Clinic |
| India | Dr. Kabir's Clinic |
| India | Dr. Kolke Clinic |
| India | Dr. Kothiwale V.A. Clinic |
| India | Dr. M. K. Mukhopadhyay's Diabetic Clinic |
| India | Dr. M.V. JALI Chief Diabetologist Clinic |
| India | Dr. Meena Chhabra Clinic |
| India | Dr. Muskara Clinic |
| India | Dr. P.K. Goyal's Clinic |
| India | Dr. Panikar's Speciality Care Centre |
| India | Dr. Paras Gangwal Clinic |
| India | Dr. Prasad's Clinic |
| India | Dr. Rahman's Clinic |
| India | Dr. Rajeev Bansal's Cardio Diabetes Clinic |
| India | Dr. Rajendra Kumar Clinic |
| India | Dr. Rajesh G. Nair Clinic |
| India | Dr. Rudredu Bhattacharya Clinic |
| India | Dr. (Mrs.) Sagarika Mukherjee's Private Clinic |
| India | Dr. Samit Ghosal's Clinic |
| India | Dr. Sandip Mandal's Clinic |
| India | Dr. Sarfaraj Majid Clinic |
| India | Dr. Shaibal Chakraborty's Clinic |
| India | Dr. Shalini Jaggi Clinic |
| India | Dr. Venugopala D's clinic |
| India | Dr. Hotwani's Clinic |
| India | Dr. Parminder Singh's Clinic |
| India | East Coast Diabetes Centre-3 |
| India | Eastern Diagnostic & Medical Centre |
| India | Endocrine and Diabetes Care |
| India | Erode Diabetes Foundation |
| India | Escorts Fortis Hospital and Research Centre Ltd |
| India | ESIC Hospital, KK Nagar Chennai |
| India | Falby Multi Speciality Clinic |
| India | Gomber Medicare |
| India | Gujarat Endocrine Centre |
| India | H.P. Clinic |
| India | The Healing Touch Clinic |
| India | Heart and Diabetic Clinic |
| India | Indraprastha Apollo Hospital |
| India | Indus Diabetes and Obesity Centre |
| India | Inlaks and Budhrani Hospital |
| India | Instride |
| India | Integral Coach Factory Hospital |
| India | IPGMR&E, SSKM Hospital |
| India | Iqbal Dakhni Memorial Centre |
| India | J.K. Institute |
| India | Jagjivan Ram Hospital |
| India | JIP Diabetic Care Centre |
| India | Joshi Diabetes and Heart Clinic |
| India | Jothydev's Diabetes Research Centre |
| India | JR's Diabetes and Wellness Center |
| India | K.R. Hospital |
| India | Kannur Medical College Hospital |
| India | Kelavkar Medical Centre |
| India | KGN Diabetes and Endocrinology Centre |
| India | KOAI Diabetes Speciality Centre And Hospital |
| India | Kripalani Hospital |
| India | Latur Diabetes Care |
| India | Life Aid Clinic |
| India | Lina Diabetes Clinic |
| India | LLRM Medical College Meerut |
| India | Prof. M. Viswanathan Diabetes Research Centre |
| India | Madonna Hospital |
| India | Madonna Hospital And Research Centre |
| India | Manipal Hospital |
| India | Max Hospital |
| India | Max Super Speciality Hospital |
| India | MBPT Hospital |
| India | Meenakshi Mission Hospital and Research Centre |
| India | Modern Hospital |
| India | Monicka Diabetes Centre |
| India | Najath Super Speciality Hospital |
| India | Nalam Hospital |
| India | Oxybliss Cardio-Diabetic Care and Research Centre |
| India | Palani Kumaran Diabetes Hospital |
| India | Park Hospital |
| India | Pondicherry Diabetes Specialities Centre |
| India | Poona Diabetes Centre |
| India | Poorni Devi Kanoria Diabetes Clinic |
| India | Prabhakar Kore's KLES Hospital |
| India | Prarthana Clinic & Diabetes Care Centre |
| India | Pristine Diabetes Centre |
| India | Priya Nursing Home |
| India | Rabindranath Tagore Hospital |
| India | Relief Poly Clinic |
| India | Risk Care Hospital and ICCU |
| India | Rithika Diabetes And Endocrine Centre |
| India | S.K. Diabetes Research & Education Centre |
| India | Salem Gopi Hospitals (P) Ltd. |
| India | Satyam Medical Centre |
| India | Savita Clinic |
| India | SG Diabetes Centre |
| India | Shakthi Clinic |
| India | Sharada Medical Centre |
| India | Shatabdi Hospital |
| India | Shivajoyti Clinic |
| India | Shreya Diabetes Care Centre |
| India | Shreyas Clinic |
| India | Shrimahavir Clinic |
| India | SIR Ganga Ram Hospital |
| India | Dr. S.K. Singh's Clinic |
| India | South City Clinic |
| India | SPT Hospitals |
| India | Sri Raghavendra Nursing Home, Diabetes Care Centre |
| India | STAR Hospital |
| India | Sudeep Diabetes Care Centre |
| India | Sun Valley Hospital & Diabetes Research Center |
| India | Sunshine Diagnostic Centre |
| India | Synergy Hospital |
| India | Thakur Hospital |
| India | Tirupati Poly Clinic |
| India | Tongaonkar Hospital |
| India | Trish Dil Diabetic Care |
| India | Tulasi Diabetic Clinic |
| India | Udayam |
| India | Upchaar |
| India | Vasavi Poly Clinic |
| India | Venkata Kripa Diabetic Centre |
| India | Vijaya Clinic-Diabetic Care Center |
| India | Vijaya Diabetes And Speciality Hospital |
| India | Vijayratna Diabetes Diagnosis and Treatment Centre |
| India | Vikram Hospital Private Limited |
| India | Zoom Health Care |
| Indonesia | Bros Hospital |
| Indonesia | Cardio Diabetes Group (CDG) Clinic |
| Indonesia | Dr. Roeslina Herawati Clinic |
| Indonesia | Gading Pluit Hospital |
| Indonesia | Kalimosadha Clinic |
| Indonesia | Mandiri Clinic |
| Indonesia | Mitra Kemayoran Hospital |
| Indonesia | Persahabatan Hospital |
| Indonesia | Puri Cinere Hospital |
| Indonesia | Santosa Hospital |
| Indonesia | SS Diabetes Care Clinic |
| Korea | Baoro Clinic |
| Korea | Bundang 21st Century Clinic |
| Korea | Hallym University Dongtan Sacred Heart Hospital |
| Korea | Kyunghee University Medical Center |
| Korea | Lee Won Pyo Clinic |
| Korea | The Catholic University of Korea, Bucheon St. Mary’s Hospital |
| Korea | The Catholic University of Korea, Seoul St. Mary's Hospital |
| Malaysia | Department of Medicine, University of Malaya |
| Malaysia | Department of Primary Care Medicine, University of Malaya |
| Malaysia | Horeb Services SDN BHD |
| Malaysia | Pantai Hospital Kuala Lumpur |
| Philippines | Activeone Health Inc. |
| Philippines | AFPMC Diabetes Clinic |
| Philippines | Air Force General Hospital |
| Philippines | Asian Hospital and Medical Center |
| Philippines | Associated Doctors Clinic |
| Philippines | Baclaran Medical Doctors Clinic |
| Philippines | Baguiio Kidney Care Clinic Baguio General Hospital |
| Philippines | Baguio General Hospital and Medical Center |
| Philippines | Bangko Sentral ng Pilipinas |
| Philippines | Bauang Medical and Diagnostic Center |
| Philippines | Bautista Hospital Diabetes Center |
| Philippines | Belocura Diabetes and Medical Clinic |
| Philippines | Benguet General Hospital Diabetes Clinic |
| Philippines | Bethany Hospital |
| Philippines | Binan Doctors Hospital |
| Philippines | Brokenshire Hospital |
| Philippines | Bureau of Corrections, New Bilibid Prison Hospital |
| Philippines | Cagayan De Oro Medical Center |
| Philippines | Calamba Medical Center |
| Philippines | Cardinal Santos Medical Center |
| Philippines | CDC San Jose |
| Philippines | Center for Diabetes Care |
| Philippines | Chinese General Hospital |
| Philippines | Christian Medical Specialist Clinic, Inc. |
| Philippines | Clinica Familia |
| Philippines | Clinica Tenorio |
| Philippines | Coca Cola Bottles Philippines Medical Clinic |
| Philippines | Court Of Appeals Clinic |
| Philippines | Decena General Hospital |
| Philippines | Dela Salle Medical Center |
| Philippines | Department Of Agrarian Reform |
| Philippines | Diabetes Clinic, Medical Lane |
| Philippines | Dr. JS Aspiras Med Clinic |
| Philippines | Dr. Gil Decierto's Medical Clinic |
| Philippines | Dr. Marcelino Salango Clinic |
| Philippines | Dr. Pablo O. Torre Memorial Hospital |
| Philippines | Feu Nrmf Medical Center |
| Philippines | Fortmed Medical Clinics Sta Rosa |
| Philippines | Fortune Care Clinic Angeles City |
| Philippines | Fortune Care HMO |
| Philippines | Fortune Care Inc |
| Philippines | Global Care Medical Group Specialists Inc. |
| Philippines | Green Pasteur Diabetes Specialty Clinic |
| Philippines | Health Serv Los Banos Medical Center |
| Philippines | Healthway Medical Alabang Tower Center |
| Philippines | Holcim Clinic |
| Philippines | House of Representatives |
| Philippines | Ilocos Traninc and Recionnaz Medical Center |
| Philippines | Intellectual Property Office |
| Philippines | International Health Aide Diagnostic Services Inc. |
| Philippines | La Union Medical Center |
| Philippines | La Union Medical Diagnostic Center |
| Philippines | Las Pinas City Medical Center |
| Philippines | Las Pinas Doctors Hospital |
| Philippines | Las Pinas General Hospital and Satellite Trauma Center |
| Philippines | Lorma Medical Center |
| Philippines | Lucena United Doctors Hospital |
| Philippines | Lucena United Doctors Hospital and Medical Center |
| Philippines | Luzon Medical Center |
| Philippines | Ma Estreus General Hospital |
| Philippines | Makati Health Department Employees Clinic |
| Philippines | Makati Medical Center |
| Philippines | Mallari Family Care Clinic |
| Philippines | Mamra Doctors Hospital |
| Philippines | Manila Doctor's Hospital |
| Philippines | Maniva Naval Hospital |
| Philippines | Mdi Sinai |
| Philippines | Medical Center Manila |
| Philippines | Metro Urdaneta Diagnostics Specialist Center |
| Philippines | Metropolitan Hospital |
| Philippines | Molino Doctors Hospital |
| Philippines | Montemayoris Medical Clinic |
| Philippines | Mother of Grace Poly Clinic |
| Philippines | Mother Theresa of Calcutta Medical Center |
| Philippines | MPC Clinic |
| Philippines | Municipal Health Office |
| Philippines | Notre Dame De Chatres Hospital |
| Philippines | Notre Dame Hospital |
| Philippines | Notre Damf De Chartres Hospital |
| Philippines | Ombudsman Clinic |
| Philippines | OPD 1 Family Medicine Lorma Medical Center |
| Philippines | OPD II Lorma Medical Center |
| Philippines | Our Lady of the Pillar Medical Center |
| Philippines | Paranaque Doctors Hospital |
| Philippines | PCU University Clinic |
| Philippines | PDIC |
| Philippines | Perpetual Help Medical Center - Binan |
| Philippines | Phil Nikkei Jin Kai Clinic |
| Philippines | Philippine Society of Endocrinology and Metabolism, Inc |
| Philippines | Pines City Aocrors Hospital |
| Philippines | PLDT Clinic Makati |
| Philippines | Pnoc Alternative Clinic |
| Philippines | Premiere Medical Center |
| Philippines | Provincial Health Office |
| Philippines | Rhu Agoo La Union |
| Philippines | Rivera Diabetes Clinic |
| Philippines | Rivera Medical Center |
| Philippines | Rosmed Diagnostic and Specialists Centre |
| Philippines | Salango Medical Clinic |
| Philippines | San Pablo Doctor's Hospital |
| Philippines | Senor Sto. Nino Hospital |
| Philippines | Shell Philippines |
| Philippines | St. Dominic Medical Center |
| Philippines | St. Louis Univ. Hospital of the Sacren Heart |
| Philippines | St. Frances Cabrini Medical Center and Cancer Ins. |
| Philippines | Sta Rosa Medical Center |
| Philippines | Supreme Court of the Phillipines Clinic |
| Philippines | Tarlac State University Medical Clinic |
| Philippines | The Health Cube |
| Philippines | Tokyo Health Link |
| Philippines | Trece Martires Provincial Hospital |
| Philippines | UERM Hospital |
| Philippines | University of Sto Tomas Hospital |
| Philippines | UPMC - FMAB |
| Philippines | Valdez Santos Clinic |
| Philippines | Wellcare Clinic |
| Singapore | AMPM Family Clinic & Surgery Pte Ltd |
| Singapore | Bedok Clinic & Surgery |
| Singapore | Bedok Life Clinic |
| Singapore | Han Clinic & Surgery Pte Ltd |
| Singapore | Healthway Tampines Clinic |
| Singapore | Ho Medical Centre |
| Singapore | Hong Family Clinic |
| Singapore | Lee Clinic (Clementi) |
| Singapore | Medical Centre @Commonwealth |
| Singapore | Medico Clinic & Surgery |
| Singapore | New Town Clinic Pte. Ltd. |
| Singapore | Newcastle Clinic |
| Singapore | Public Medical Clinic & Surgery |
| Singapore | Singapore General Hospital |
| Singapore | Tan Medical Clinic Pte. Ltd. |
| Singapore | Tham Clinic Singapore Pte. Ltd. |
| Taiwan | Changhua Christian Hospital |
| Taiwan | Chia Yi Christian Hospital |
| Taiwan | JOY Clinic |
| Taiwan | National Cheng Kung University Hospital |
| Taiwan | National Taiwan University Hospital |
| Taiwan | Taichung Veterans General Hospital |
| Taiwan | Taipei Veterans General Hospital |
| Taiwan | Tri-Service General Hospital |
| Thailand | Rajavithi hospital |
| Thailand | Sanpasitthiprasong Hospital |
| Thailand | Srinakharinwirot University |
| Thailand | Thammasat University Hospital |
| Thailand | Thepatarin General Hospital |
| Vietnam | 115 Hospital |
| Vietnam | An Binh Hospital |
| Vietnam | An Sinh Hospital |
| Vietnam | Au Lac clinic |
| Vietnam | Bach Mai Hospital |
| Vietnam | Diabetes Clinic |
| Vietnam | Doctor Nga's clinic |
| Vietnam | Dong Do Heart Hospital |
| Vietnam | Far East Medical Vietnam Limited |
| Vietnam | HCMC University of Pharmaceutical and Medicine |
| Vietnam | Heart Institute Hospital |
| Vietnam | Hoan My Hospital |
| Vietnam | Huynh Tan Dat's Clinic |
| Vietnam | Lam Van Hoang's Clinic |
| Vietnam | Medical University Hospital |
| Vietnam | Noi Tiet (National Endocrinology) Hospital |
| Vietnam | National Geriatric Hospital |
| Vietnam | Nguyen Trai Hospital |
| Vietnam | Nguyen Tri Phuong Hospital |
| Vietnam | Nhan Dan Gia Dinh |
| Vietnam | Phan Huu Hen Clinic |
| Vietnam | PHC Clinic |
| Vietnam | Tam Duc Hospital |
| Vietnam | Thai Ha Clinic |
| Vietnam | Thanh Hoa's Clinic |
| Vietnam | Thong Nhat Hospital |
| Vietnam | Tran Van Hai's Clinic |
| Vietnam | Trieu An Hospital |
| Vietnam | Trung Vuong Hospital |
| Vietnam | Van Hanh Hospital |
| Vietnam | Viet Nam National Heart Institute |
| Vietnam | VIP 12 Clinic |

Table S2. Interaction effects of family history of diabetes and self-management on cardiometabolic risk factors

|  | Hyperglycemia† | Hypertension‡ | Dyslipidemia§ | ‘A' goal achieved† | ‘B' goal achieved† | ‘C' goal achieved† | ≥ 2 ‘ABC’ goals achieved† |
| --- | --- | --- | --- | --- | --- | --- | --- |
|  | aOR (95% CI) | aOR (95% CI) | aOR (95% CI) | aOR (95% CI) | aOR (95% CI) | aOR (95% CI) | aOR (95% CI) |
| FamH+ × Adequate physical activity | 0.91 (0.84 - 0.98)* | 0.91 (0.85 - 0.98)* | 0.96 (0.87 - 1.05) | 1.06 (0.99 - 1.14) | 1.09 (1.02 - 1.16)* | 1.09 (1.02 - 1.16)* | 1.02 (0.95 - 1.10) |
| FamH+ × Adherence to balanced diet | 0.92 (0.85 - 0.99)* | 0.90 (0.84 - 0.97)** | 0.97 (0.88 - 1.06) | 1.07 (1.00 - 1.15)* | 1.03 (0.96 - 1.10) | 1.06 (0.99 - 1.13) | 1.09 (1.02 - 1.17)* |
| FamH+ × Never/ ex-smoker | 0.75 (0.66 - 0.84)*** | 0.76 (0.68 - 0.85)*** | 0.79 (0.68 - 0.93)** | 1.22 (1.10 - 1.36)*** | 1.39 (1.26 - 1.54)*** | 1.21 (1.09 - 1.34)*** | 1.26 (1.13 - 1.41)*** |
| FamH+ × Never/ occasional drinker | 0.82 (0.75 - 0.90)*** | 0.88 (0.81 - 0.96)** | 0.99 (0.88 - 1.11) | 1.17 (1.08 - 1.27)*** | 1.31 (1.21 - 1.42)*** | 1.15 (1.07 - 1.24)*** | 1.22 (1.13 - 1.33)*** |
| FamH+ × SMBG | 1.06 (0.98 - 1.16) | 1.07 (0.99 - 1.16) | 1.06 (0.95 - 1.18) | 1.01 (0.94 - 1.09) | 0.94 (0.87 - 1.01) | 1.00 (0.93 - 1.07) | 0.97 (0.89 - 1.05) |
| FamH+ × ≥3 favourable behaviors | 0.94 (0.84 - 1.04) | 0.90 (0.82 - 0.98)* | 0.94 (0.83 - 1.08) | 1.10 (1.00 - 1.20)* | 1.20 (1.10 - 1.31) *** | 1.18 (1.08 - 1.28) *** | 1.19 (1.08 - 1.30) *** |

Footnotes: FamH = family history of diabetes, aOR = adjusted odds ratio, 95% CI = 95% confidence interval, SMBG = self-monitoring of blood glucose.

Adequate physical activity refers to exercise for ≥30 minutes with at least 3 times weekly.

Favorable behaviors include self-monitoring of blood glucose, the use of alcohol and tobacco, adherence to a balanced diet, and physical activity.

All models were adjusted for age, sex, education (middle school and above *vs.* primary school or below), employment (worker *vs.* non-workers), drug use (oral glucose lowering drug, insulin, lipid regulating drug, blood pressure lowering drug, and renin-angiotensin system inhibitors), duration of diabetes, year of enrolment, and country or region of recruitment.

†Hyperglycemia = HbA_1c_ > 7% (53 mmol/mol) or fasting blood glucose > 7 mmol/L

‡Hypertension = Blood pressure ≥ 140/90 mmHg or on any blood pressure lowering drugs.

§Dyslipidemia= LDL-C ≥ 2.6 mmol/L, HDL-C < 1 mmol/L, triglycerides ≥ 2.3 mmol/L, or on any lipid‐lowering drugs

***p < 0.001, **p < 0.01, *p < 0.05.

Figure S1. Conceptual framework of the complex interactions between family history and behavioral factors on development of type 2 diabetes and cardio-metabolic control.


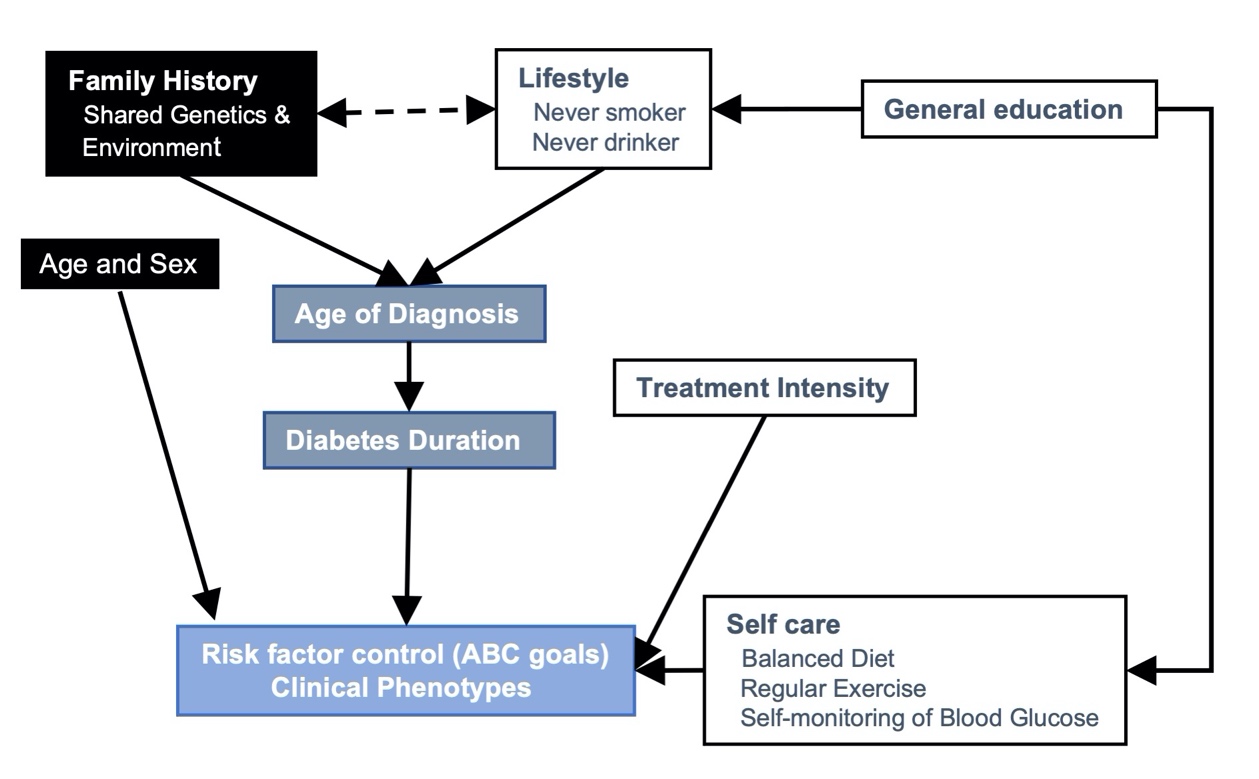


Footnotes: ABC goals refer to HbA_1c_ <7% (53 mmol/mol) (A), blood pressure <130/80 mmHg (B) and LDL-C <2.6 mmol/L (C). Black boxes indicate non-modifiable factors including family history, age, gender and disease duration. White boxes indicate modifiable factors include lifestyles, general education, self-care, and medications. Modifiable and non-modifiable factors may interact to contribute to the control of ABC goals.

Figure S2. Kaplan–Meier estimate of cumulative proportion for age at diagnosis among 8,556 patients with type 2 diabetes diagnosed within 1 year prior to assessment

Footnotes: FamH = family history of diabetes (father, mother and/or siblings). Healthy lifestyles include adequate physical activity (30 minutes at least 3 times weekly), adherence to a balanced diet, never or occasional alcohol drinker, and never or ex-smoker.

The figure shows the mean age at diagnosis (years) in FamH- group with <2 healthy lifestyles vs. FamH- group with ≥2 healthy lifestyles vs. FamH+ group with <2 healthy lifestyles vs. FamH+ group with ≥2 healthy lifestyles: [53.9, 95% CI 53.1-54.7] *vs.* [57.0, 95% CI 56.6-57.3] *vs.* [49.9, 95% CI 49.3-50.5] *vs.* [52.3, 95% CI 52.0-52.6].
